# *Cryptococcus* genetic diversity and mixed infections in Ivorian HIV patients: A follow up study

**DOI:** 10.1371/journal.pntd.0007812

**Published:** 2019-11-18

**Authors:** Fulgence Kondo Kassi, Pascal Drakulovski, Virginie Bellet, Frédéric Roger, Amélie Chabrol, Donika Krasteva, Adama Doumbia, Roland Landman, Aka Kakou, Jacques Reynes, Eric Delaporte, Hervé Eby Ignace Menan, Sébastien Bertout

**Affiliations:** 1 Université Félix Houphouet-Boigny, Unité des Sciences Pharmaceutiques et Biologiques, Abidjan, Côte d’Ivoire; 2 Laboratoire de Parasitologie et Mycologie Médicale, IRD UMI 233, INSERM U1175, Université de Montpellier, Unité TransVIHMI, Montpellier, France; 3 Service de Maladies Infectieuses et Tropicales, CH Sud Francilien, Corbeil, France; 4 Institut de Médecine et Epidémiologie Appliquée (IMEA), Fondation Léon M’Ba, Paris, France; 5 Service des Maladies Infectieuses et Tropicales, CHU Treichville, Abidjan, Côte d’Ivoire; 6 CHU Gui de Chauliac, Service des Maladies Infectieuses et Tropicales, IRD UMI 233, INSERM U1175, Université de Montpellier, Unité TransVIHMI, Montpellier, France; 7 TransVIHMI/INSERM1175, Institut de Recherche pour le Développement (IRD) and University of Montpellier, Montpellier, France; 8 Diagnostic and Research Center on AIDS and Other Infectious Diseases (CeDReS), Abidjan, Côte d'Ivoire; National Institute for Communicable Diseases, Johannesburg, South Africa, SOUTH AFRICA

## Abstract

Genetic diversity analyses were performed by sero-genotyping and multi-locus sequence typing on 252 cryptococcal isolates from 13 HIV-positive Ivorian patients followed-up for cryptococcal meningitis. Antifungal susceptibility analyses were performed according to the CLSI M27A3 method. The majority (67.8%) of the isolates belonged to the *Cryptococcus neoformans* (serotype A) species complex, with 93% being VNI and 7% being VNII. *Cryptococcus deuterogattii* VGII (serotype B) represented 16.7% of the strains, while *C*. *neoformans*/*C*. *deneoformans* VNIII (serotype AD) hybrids accounted for 15.1% of the strains. One strain (0.4%) was not identifiable. Nine different sequence types (STs 5, 6, 23, 40, 93, 207, 311, and a new ST; 555) were identified in the *C*. *neoformans* population, while the *C*. *deuterogattii* population comprised the single ST 173. The distribution of the strains showed that, while the majority of patients (9/13) harboured a single sequence type, 4 patients showed mixed infections. These patients experienced up to 4 shifts in strain content either at the species and/or ST level during their follow-up. This evolution of diversity over time led to the co-existence of up to 3 different *Cryptococcus* species and 4 different ST within the same individual during the course of infection. Susceptibility testing showed that all strains were susceptible to amphotericin B while 3.6% of them had a none-wild type phenotype to 5-flucytosine. Concerning fluconazole, 2.9% of *C*.*neoformans* serotype A strains and 2.4% of *C*. *deuterogattii* had also respectively a none-wild type phenotype to this molecule. All *C*. *neoformans* x *C*. *deneoformans* serotype AD hybrids had however a wild type phenotype to fluconazole. The present study showed that mixed infections exist and could be of particular importance for care outcomes. Indeed, (i) the different *Cryptococcus* species are known to exhibit different virulence and different susceptibility patterns to antifungal drugs and (ii) the strains genetic diversity within the samples may influence the susceptibility to antifungal treatment.

## Author summary

Cryptococcal meningitis is a neglected fungal disease responsible for 181 000 deaths worldwide in 2014, with 75% of deaths occurring in sub-Saharan Africa. Cryptococcal meningitis is caused by environmental yeasts belonging to the *Cryptococcus neoformans*/*Cryptococcus gattii* species complexes. The evolution of the diversity of the yeast populations within the patients and during the course of treatment is poorly understood. Indeed, it was believed for a long time that infections were of a single strain type. It was only recently that the complexity of the yeast diversity during infection began to be assessed. Here, the researchers evaluated the diversity of the *Cryptococcus* population within Ivoirian patients. The purpose was to generate data about the overall diversity of such yeast in Western Africa where the data are scarce and to better understand the evolution of the pathogen populations during patient follow-up. This last point is particularly important because some species are more virulent or naturally more resistant to antifungal treatments and could be an issue in the case of relapses during care protocols.

## Introduction

The *Cryptococcus neoformans* and *Cryptococcus gattii* yeast species complexes are the aetiological agents of cryptococcosis [1], a fungal disease affecting mainly immunocompromised hosts [2]. The course of this disease leads in most clinical cases to cryptococcal meningitis (CM), which is often lethal. CM is mainly acquired through inhalation of dehydrated yeast cells and spores from environmental sources, including pigeon excreta, plant debris and decaying wood [3–4]. *Cryptococcus* may cause pneumonia and is able to disseminate to the central nervous system (CNS), where it infects the brain parenchyma [5]. In 2014, annual fatalities from CM were estimated to be 181 100 deaths globally, with 135 900 deaths occurring in sub-Saharan Africa [2]. Globally, CM results in 15% of AIDS-related mortality, with sub-Saharan Africa bearing the greatest burden of this disease. CM is an excellent metric of HIV (human immunodeficiency virus) treatment programme failure [6]. Indeed, the frequent final outcome in a failed cascade of HIV care is the development of CM because of late diagnosis, no HAART (highly active antiretroviral therapy) access, care breakdown, and virological failure of HAART [2]. Furthermore, the increasing number of people living with other immunodeficiencies, including transplant and cancer patients, represents a growing population at risk for CM [7].

Several molecular methods have been used for the detection of specific genetic sequences of the *C*. *neoformans* and *C*. *gattii* species complexes. The most commonly used approaches are PCR fingerprinting, PCR-RFLP of the *URA5* gene, AFLP and multi-locus sequence typing (MLST) [8–11]. Initially restricted to *C*. *neoformans* (with two varieties *grubii* and *neoformans*)[12,13] and *C*. *gattii* and 2 serotypes [14,15], the taxonomy of *C*. *neoformans/C*. *gattii* species complex was revised recently by Hagen *et al*. due to these methods. The taxonomy now contains seven species and nine genotypes based on phylogenetic and genotypic studies. *C*. *neoformans* variety *grubii* has been renamed *C*. *neoformans* (serotype A, genotype AFLP1/VNI, AFLP1A/VNB/VNII and AFLP1B/VNII). *C*. *neoformans* var. *neoformans* has been renamed to *C*. *deneoformans* (serotype D, genotype AFLP2/VNIV). Within the *C*. *gattii* species complex, five distinct species have been described, namely, *C*. *gattii* (serotype B, genotype AFLP4/ VGI), *C*. *bacillisporus* (serotype C, genotype AFLP5/VGIII), *C*. *deuterogattii* (serotype B, genotype AFLP6/VGII), *C*. *tetragattii* (serotype C, genotype AFLP7/VGIV) and *C*. *decagattii* (serotype B, AFLP10/VGIV) [16, 17]. The precise mechanisms that determine the prevalence of the various cryptococcal species are still unknown but seem to be associated with host status, as well as geographical and environmental factors [18]. *Cryptococcus neoformans genot*ypes VNI and VNII are widely distributed throughout the world and are strongly associated with urban areas and bird guano as well as several trees [19], with VNI being the major cause of CM in HIV-infected individuals [20,21]. VNB genotypes have been identified in South Africa, Botswana, DRC, Rwanda and Zambia, where they represent up to 30% of the isolates and are associated with an arboreal environment [20,22]] but also in few other countries [23]. The *C*. *gattii* complex species were initially found in tropical and subtropical areas, but currently, the geographic distribution of *C*. *gattii* infections has expanded to temperate climate regions, including Canada and the USA [11]. Clinical manifestations in patients with *C*. *gattii* infections tend to be more severe than those with *C*. *neoformans*. With *C*. *gattii* infection, cerebral involvement causes more hydrocephalus, focal CNS signs, ataxia, hearing loss, altered mentation, and neurological sequelae. Simultaneous pulmonary involvement is also observed, and cryptococcomas are associated with a prolonged clinical course and slow response to therapy [24].

Currently, therapeutic management of cryptococcal meningitis (CM) in severely immunosuppressed hosts is formalized around the concepts of induction, consolidation, and maintenance phases. The therapeutic regimen currently recommended by the WHO for the control of CM in HIV patients, particularly during consolidation and maintenance phase, uses a combination of either amphotericin B and 5-flucytosine (5FC) or fluconazole and 5FC, depending on the access to these drugs. In sub-Saharan Africa, amphotericin B and 5FC are rarely or not available. Consequently, fluconazole (FCZ) is the most commonly administered drug for cryptococcosis treatment in this region, with up to 80% of infections treated by FCZ monotherapies [25]. This limited drug arsenal leads to variable prognoses and poor survival outcomes [26]. Furthermore, different antifungal susceptibility patterns have been observed among the cryptococcal species. In general, the *C*. *gattii* species complex shows higher minimum inhibitory concentrations (MICs) for azoles than isolates from the *C*. *neoformans* species complex [27, 28], making patient care even more difficult in high-burden low-resource countries.

The authors previously reported a high genetic diversity and antifungal susceptibility of *C*. *neoformans/C*. *gattii* species complexes from clinical sources in Yaoundé, Cameroon and Abidjan, and Ivory Coast and showed that *C*. *neoformans* (A, AFLP1/VNI) is widespread in the environment and is associated with the majority of cases of cryptococcosis in Ivory Coast [29–31]. During these studies, the authors demonstrated that some patients suffered concurrent infections by different sero-genotypes, including mixed infections by two different *Cryptococcus* species, *C*. *neoformans* AFLP1/VNI and *C*. *deuterogattii* AFLP6/VGII [29]. Thus, the possibility of mixed infection must be considered for the management of cryptococcosis. Detection of such infections in samples without follow-up was possible by analysing multiple isolates instead of a single isolate for each clinical sample.

However, little is known about *Cryptococcus* population diversity evolution in the same HIV-positive patient with cryptococcal disease in follow-up samples over time. The present study analysed the epidemiology of strains from follow-up samples in each patient and between several patients by the same multiple isolate methodology. In addition, the genetic diversity of the *Cryptococcus species complex* from the current cohort was compared by MLST typing with that of isolates collected from other countries. Susceptibilities of isolated strains to fluconazole and flucytosine, two drugs used during patient infection, and against amphotericin B, the gold standard treatment, were assessed.

## Methods

### Prospective study protocol: Patient inclusion and strain identification

This prospective study was performed as an ancillary study to the ANRS 12257 Study [32] at the teaching hospital of Treichville, Infectious and Tropical Diseases Unit (SMIT) of Abidjan, Ivory Coast, between May 2014 and September 2015. The included patients were HIV positive, and none had received a systemic antifungal treatment prior to the study. After inclusion, patients received fluconazole (FCZ) (1600 mg per day) for 14 days in combination with flucytosine (100 mg/kg per day) followed by FCZ alone (800 mg per day) for up to 10 weeks of follow-up and FCZ (200 mg/day) until immunity restoration Antiretroviral treatment with emtricitabine, tenofovir and efavirenz started on Day 15 (D15). CM was confirmed by the identification of *Cryptococcus* in CSF (cerebrospinal fluid) using direct examination with China ink by detection in CSF of the cryptococcal antigen latex agglutination slide tests with Pastorex Crypto Plus kit (Bio-Rad, Marnes-la-Coquette, France) and by positive culture on Sabouraud’s medium. The identification of each strain after culturing was performed via a positive urea–indole test and the commercial identification kit ID32C (Biomerieux, Marcy-l’Étoile, France). The CSF was recovered at regular intervals for each patient: on the first day (D0), the 7^th^ day (D7), the 14^th^ day (D14), the 28^th^ (D28), the 10^th^ week (W10) and more if needed. One patient was resampled in the 26^th^ week following a relapse. For each patient, the entire culture and five isolates of *Cryptococcus* were recovered as previously described [29–31, 33]. Phenotypic characterization of the *Cryptococcus* species was achieved by chemotyping in L-canavanine-glycine-bromothymol blue (CGB) agar. CGB agar was used to differentiate *C*. *neoformans* complex species and *C*. *gattii* complex species as described previously. The blue colour of glycine assimilation on CGB agar indicated a positive reaction caused by the *C*. *gattii* species complex, whereas the *C*. *neoformans* species complex did not cause a colour change [34].

Demographic, clinical, biological and therapeutic data were collected using a structured form.

### Patients, isolates and strains

On 32 HIV-positive patients with cryptococcal meningitis included in the ANRS 12257 study at Treichville site, 13 patients with at least two positive cultures between D0 and W24 were included in our study for a total of 42 samples and thus 252 isolates.

A set of standard laboratory reference strains representing each of the eight major molecular types were used for molecular typing: WM148 (= CBS10085 = ATCC MYA-4564, VNI, serotype A), WM626 (= CBS10084 = ATCC MYA-4565, VNII, serotype A), WM628 (= CBS10080 = ATCC MYA4566, VNIII, serotype AD), WM629 (= CBS10079 = ATCC MYA-4567, VNIV, serotype D), WM179 (= CBS10078 = ATCC MYA-4560, VGI, serotype B), WM178 (= CBS10082 = ATCC MYA-4561, VGII, serotype B), WM161 (= CBS10081 = ATCC MYA-4562, VGIII, serotype B) and WM779 (= CBS10101 = ATCC MYA-4563, VGIV, serotype C)[9].

### DNA extraction

Genomic DNA was extracted for each strain and entire culture using extraction kit NucleoSpin blood quick (Macherey-Nagel Gmb and Co. KG, Duren, Germany) with modifications as previously described [29]. One aliquot was used for each of the experiments described in this study.

### Molecular typing

#### Multiplex PCR serotyping

To determine the molecular type, four primers designed for cloning *LAC1* and a pair of primers for *CAP64* [35, 36] were used in a slightly modified method as previously described [29, 30].

#### URA5-RFLP PCR genotyping

PCR-RFLP analyses were performed using the URA5 and SJ01 primers [29, 30]. The reaction conditions were as follows: initial denaturation (94°C, 2 minutes), 35 cycles of denaturation (94°C, 45 seconds), annealing (61°C, 1 minute) and extension (72°C, 2 minutes), and a final extension cycle (72°C for 10 minutes). Ten microliters of each PCR product was double digested using *Sau96I* (15 U) and *Hhal* (15 U) for 5 hours at 37°C, and the digested fragments were visualized on 1.5% agarose gels stained with ethidium bromide [9]. Migration patterns were captured with an Ingenius LR apparatus (Syngene, UK) Molecular profiles obtained *via* PCR fingerprinting were analysed based on the presence or absence of readily apparent and well-defined bands in the digitized gel images with GeneSnap and Genetool software (Syngene, UK) and integrated in a database using GeneDirectory software (http://www.syngene.com/genedirectory-2/ Syngene, UK).

#### MultiLocus Sequence Typing (MLST) and analysis

The International Society for Human and Animal Mycology (ISHAM) MLST consensus schemes described for the *C*. *neoformans* and *C*. *gattii species complex* was used in this study [10]. The six genes *CAP59*, *GPD*, *LAC1*, *PLB1*, *SOD1*, *URA5* and the IGS1 region have been partially amplified [10]. PCR amplicons were purified and sequenced with forward primers by Genewiz, London, United Kingdom. Sequences were manually edited and aligned using BioEdit software [37]. Alleles types (AT) and sequences types (ST) were assigned by sequences comparisons with the *C*. *neoformans* and *C*. *gattii* databases in access at http://mlst.mycologylab.org. DnaSP 5.10 (http://www.ub.edu/dnasp/) [38] was used to determine genetic features. Minimum Spanning Tree (MST) was generated with Phyloviz 2.0 software (http://phyloviz.net/wiki/) using goeBURST algorithm. We compared allelic profiles (ST) obtained in our study among themselves. Then, we compared our allelic profiles with all other ST described for *C*. *neoformans* available on mycologylab database. The diagrams show clonal complex (CC) defined by a single locus variant (SLV) between two linked STs [39].

### Antifungal susceptibility testing

The *in vitro* susceptibility profile of *Cryptococcus* species against antifungal agents was determined using the reference broth microdilution method in accordance with document M27-A3 of the Clinical and Laboratory Standards Institute (CLSI) [40]. The final antifungal concentrations ranged from 0.125 to 16 μg mL^-1^ for amphotericin B and from 0.25 to 64 μg mL^-1^ for fluconazole and flucytosine. The minimal inhibitory concentrations (MICs) for fluconazole and flucytosine were defined as concentrations causing a 50% reduction in turbidity compared to the growth of the control at 72 hours. For amphotericin B, the MIC was defined as the concentration resulting in 100% inhibition relative to the growth of the control. *Candida krusei* ATCC 6258 and *Candida parapsilosis* ATCC 22019 were used as control strains [40].

For the *C*. *neoformans* and *C*. *gattii* species complex, no break-points are available to follow, and in this case, we used epidemiological cut-off values to discriminate wild-type strains from mutants with reduced susceptibility to some antifungals [41–44].

### Ethics statement

This study was approved by the Ethical Sciences Committees of Life and Health of the Ivory Coast (021/MSLS/CNER-kp). Written inform consent forms were signed by patients or a family member prior to the sample collection and data collected concerning them were anonymized.

### Accession numbers

Allele type sequences described in this study were previously deposited at EMBL by Beale *et al* in 2015 for South African strains [45]. The allele type sequences for Ivory Coast strains from the present study were registered on Genbank under the following accession numbers:

MN431741, MN431742, MN431743, MN431744, MN431745, MN431746, MN431747, MN431748, MN431749, MN431750, MN431751, MN431752, MN431753, MN431754, MN431755, MN431756, MN431757, MN431758, MN431759, MN431760, MN431761, MN431762, MN431763, MN431764, MN431765, MN431766, MN431767, MN431768, MN431769, MN431770, MN431771.

Correspondences for *C*. *neoformans* are the following ones:

*Cap*59 alleles 1, 2 and 7 are referenced respectively under MN431741, MN431742, and MN431743.

*GPD1* alleles 1, 3, 9 and 23 are referenced respectively under MN431745, MN431746, MN431747, and MN431748.

*IGS1* alleles 1, 10 and 14 are registered respectively under MN431750, MN431751, and MN431752 accession numbers.

*LAC1* alleles 2, 3, 5 and 8 are referenced respectively under MN431754, MN431755, MN431756, and MN431757.

*PLB1* alleles 1, 2, 4 and 11 are referenced respectively under MN431759, MN431760, MN431761, and MN431762.

*SOD1* alleles 1 and 12 are registered respectively under MN431764 and MN431765 accession numbers.

*URA5* alleles 1, 2, 4 and 5 are referenced respectively under MN431767, MN431768, MN431769, and MN431770.-

Corresponding accession numbers for *C*. *gattii* (ST 173) are: MN431744 for *Cap*59 allele 4; MN431749 for *GPD1* allele 21; MN431753 for *IGS1* allele 21; MN431758 for *LAC1* allele 4; MN431763 for *PLB1* alleles 16; MN431766 for *SOD1* allele 93 and MN431771 for *URA5* allele 2.

## Results

### Demographic characteristics of the study population

From May 2014 to December 2015, thirteen HIV-positive patients with CM were included in the study. All patients were infected with HIV type 1, except for one patient, who was infected with HIV types 1 and 2 (patient 6, S1 Table). The male/female ratio was 6/7. The mean age was 43 ± 7 years. The two major reasons for consultation were generalized prurigo (5/13) and significant weight loss (5/13). The other reasons for consultation were cerebellar toxoplasmosis, tuberculosis, fever, ophthalmic zoster, genital herpes, and furunculosis. For each patient, from the CSF, the initial culture and five separate colonies randomly collected from each initial sample were analysed. In total, the authors analysed 42 entire cultures and 210 clones for a total of 252 isolates.

At the beginning of the study, the CD4 count for each patient was <100/mm^3,^ indicating an advanced stage of HIV infection (S1 Table)

### Global species and genotype distribution

**Fig 1:**
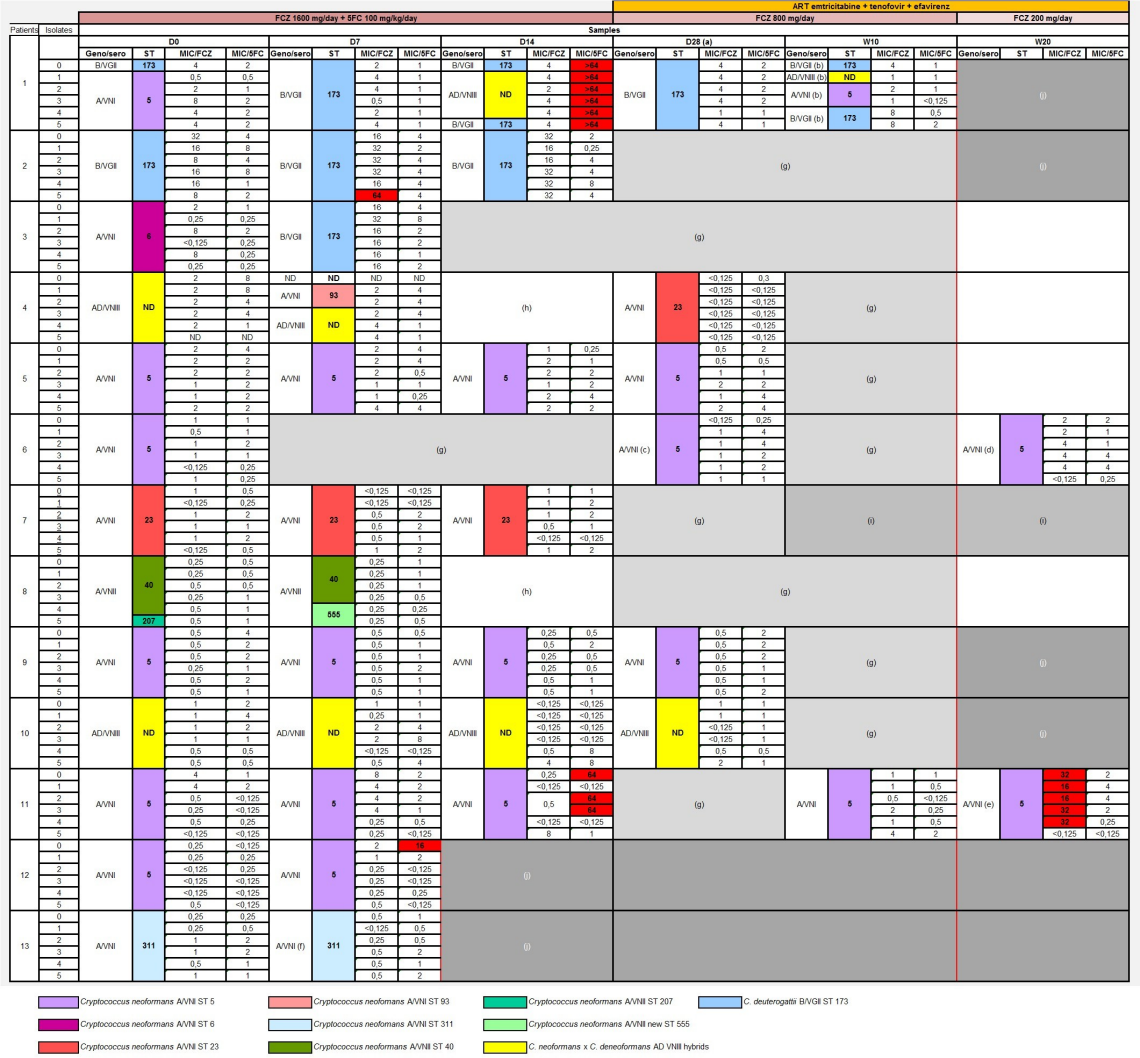
Sero-genotyping, ST characterization and MIC (μg mL^-1^) to fluconazole and flucytosine for the totality of the strains isolated during the follow up. (a) Indicate a depletive spinal tap; (b) indicate patient sampled at W9, dead at W10; (c) indicate spinal fluid positive, culture negative in Ivory Coast, positive in Montpellier, (d) indicate a patient sampled outside ANRS protocol because of relapse at W24, (e) indicate a patient sampled outside ANRS protocol with a discharge spinal tap at W26, (f) indicate a patient sampled at D10, death at D14, (g) and light grey indicate negative spinal fluid and cultures, (h) indicate positive spinal fluid, cultures positives in Ivory Coast negative in Montpellier, (i) and middle grey indicate a patient lost to follow up, (j) and strong grey indicate a deceased patient. ND shows undetermined ST. The red bar shows the ANRS 12257 Study Endpoint. MIC of the strains with a not wild type phenotype for fluconazole and flucytosine are shown in **bold and in red squares**.

Among the 252 isolates, the majority (n = 171; 67.8%) belonged to the *C*. *neoformans* (serotype A) species complex. The isolates were distributed between 159 VNI (93%) and 12 VNII (7%). *C*. *deuterogattii* VGII (serotype B) and *C*. *neoformans/C*. *deneoformans* VNIII (serotype AD) hybrids were identified in 42/252 (16.7%) and 38/252 strains (15.1%), respectively. The content of one isolate (entire culture from patient 4, sampling point D7) was not determined at sero-genotype levels due to mixed profile. This was the only unreadable sample (0.4%) (Fig 1).

### Global ST distribution

Among the *C*. *neoformans* VNI group, 5 sequence types (ST) were found: ST 5 (n = 115/159, 72.3%), ST 6 (n = 6/159, 3.8%), ST 23 (n = 24/159, 15.1%), ST 93 (n = 2/159, 1.3%) and ST 311 (n = 12/159, 7.5%) (Fig 1). In the *C*. *neoformans* VNII group, three STs were identified: ST 40 (n = 9/12, 75%), ST 207 (n = 1/12, 8.3%) and a new allelic type combination described for the first time (n = 2/12, 16.7%), deposited in the mycologylab *C*. *neoformans* database and assigned the number 555. This new ST 555 is defined by allele numbers combination 2-3-14-8-11-12-4.corresponding respectively to *CAP59*, *GPD1*, *IGS1* region, *LAC1*, *PLB1*, *SOD1* and *URA5* genes.

In the *C*. *deuterogattii* group, all the strains (n = 42/42) had the same ST 173 MLST allelic profile. STs in the *C*. *neoformans x C*. *deneoformans* VNIII hybrid group were undetermined.

In only one entire culture (patient 4, D7) the MLST profile could not be determined, most likely due to mixed profiles (Fig 1).

### Genetic polymorphism analyses

In *C*. *neoformans* group, results showed a low diversity with few polymorphic sites (between 7 to 12), low nucleotide (π < 0.003), low allelic type (*h*, 2 to 4) and low haplotype (0.158< *Hd* <0.526) diversities (Table 1).

**Table 1:** Genetics features of each locus and concatenated sequences.

Population	Locus	Length	S	*h*	*Hd*	π
*C*. *neoformans* (n = 165)	*CAP59*	560	6	3	0.447	0.0018
	*GPD1*	544	9	4	0.479	0.0022
	*IGS1*	724	12	3	0.158	0.0020
	*LAC1*	471	7	4	0.526	0.0028
	*PLB1*	533	8	4	0.464	0.0030
	*SOD1*	536	10	2	0.136	0.0026
	*URA5*	637	10	4	0.496	0.0024
	Concatenated	4005	62	8	0.536	0.0024
VN I (n = 153)	*CAP59*	560	1	2	0.362	0.0007
	*GPD1*	544	2	3	0.420	0.0008
	*IGS1*	723	10	2	0.026	0.0004
	*LAC1*	471	2	3	0.454	0.0014
	*PLB1*	533	2	3	0.382	0.0014
	*SOD1*	536	0	1	0	0
	*URA5*	637	2	3	0.420	0.0007
	Concatenated	4004	19	5	0.463	0.0007
VN II (n = 12)	*CAP59*	560	0	1	0	0
	*GPD1*	544	8	3	0.439	0.0058
	*IGS1*	722	0	1	0	0
	*LAC1*	471	0	1	0	0
	*PLB1*	533	0	1	0	0
	*SOD1*	529	0	1	0	0
	*URA5*	637	0	1	0	0
	Concatenated	3996	8	3	0.439	0.0008

Length expressed in nucleotide, polymorphic sites (S), haplotype number (*h*), haplotype diversity (*Hd*), Nucleotide diversity (π).

**Fig 2:**
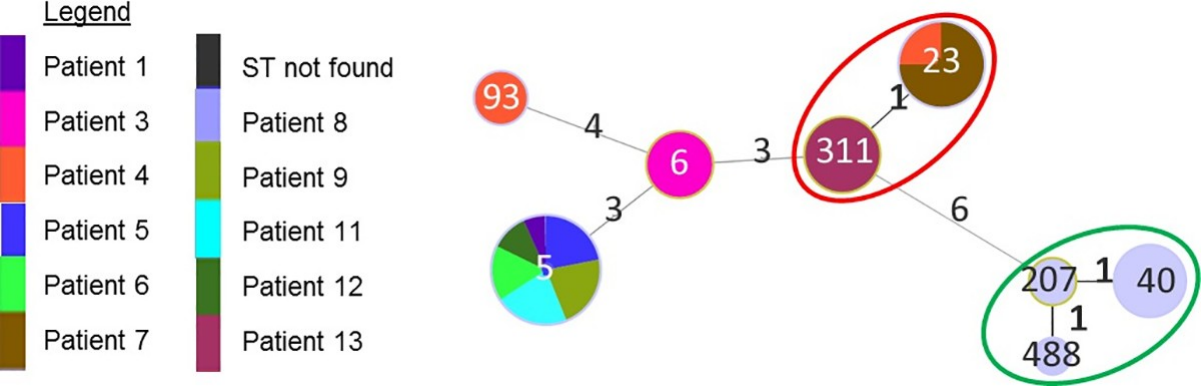
Minimum Spanning Tree showing distribution of the isolates according to patient distribution. Each patient is identified by one colour. ST forming CC are indicated by coloured circles; with a red circle showing CC including VN I isolates and a green circle showing CC including VN II isolates from the present study. Not circled STs form singletons.

When analysing only the STs found in this study, we found that they are distributed in one CC (ST 23 and 311) and three singletons (ST 5, 6 and 93) for VNI isolates. Distribution of ST for VN II isolates resulted in a single CC. (Fig 2).

The diversity of the *C*. *neoformans* population characterized here was then compared with the overall diversity in the *C*. *neoformans* database (*i*.*e*., 487 STs already present in the database + 1 new ST described in this study). Using a single locus variant (SLV) to determine the clonal complexes (CC), the 488 STs were distributed in 38 CCs and 182 singletons. The strains isolated during this study were then found in 2 distinct CCs according to their genotypes, ST 5, 6, 23, 93 and 311 for VNI isolates and ST 40, 207 and 555 for VNII isolates (S1 Fig).

Concerning the *C*. *deuterogattii* group, the population was homogenous with only one ST.

### Situation by patient

#### Species distribution by patient

Among the 13 patients studied, 11/13 were infected by *C*. *neoformans* group yeast during the course of their infection. Eight out eleven of these patients were infected only by this species (patients 5, 6, 7, 8, 9, 11, 12, and 13), while 3/11 had mixed infections involving other species (patients 1, 3 and 4) (Fig 1 and S2 Table).

*C*. *deuterogattii* was found in 3/13 patients, with one patient showing a single-species infection (patient 2) and two patients showing a mixed infection (patient 1 and 3). *C*. *neoformans x C*. *deneoformans* hybrids were found in 3/13 patients. One patient harboured a pure infection with the hybrid (patient 10), while two patients (patient 1 and 4) harboured a mixed-species infection (Fig 1 and S2 Table).

#### ST distribution by patient

Nine patients (9/13) were infected with a single ST during the course of their infection. Patients 5, 6, 9 and 11 were infected with 100% of isolates belonging to *C*. *neoformans* ST 5 while patient 7 and 13 were infected with 100% of *C*. *neoformans* ST 23 and 100% of *C*. *neoformans* ST 311 isolates respectively. Patient 2 was infected with 100% of *C*. *deuterogattii* ST 173 isolates. Patient 10 was also fully infected with strains identified as AD hybrid *C*. *neoformans x deneoformans* (Fig 1)). The absence of MLST data for serotype AD prevented us from determining the genetic links between isolates. Four patients were infected with several strains/different STs.

Concerning the four patients with mixed infections, Patient 1 presented 60% of isolates belonging to *C*. *deuterogattii* ST 173, alongside *C*. *neoformans* ST5 and *C*. *neoformans x C*. *deneoformans* hybridwhile Patient 3 was infected with 50% *C*. *deuterogattii* ST 173 and 50% *C*. *neoformans* ST 6 isolates. Patient 4 was infected with 50% *C*. *neoformans x C*. *deneoformans* hybrid, alongside *C*. *neoformans* ST 23, *C*. *neoformans* ST 93, and one isolate whose ST identification failed. Finally, patient 8 had 75% of his strains belonging to *C*. *neoformans* ST 40, alongside ST 555 and ST 207 (Fig 1).

### Evolution of diversity over time

The aforementioned patients with mixed infections (patients 1, 3, 4 and 8) showed different variations in their strain content between various follow-up dates (Fig 1).

Patient 1 presented 4 shifts in strain content from D0 to W10. At D0, his strain content was *C*. *deuterogattii* ST 173 and *C*. *neoformans* ST 5. This content changed to pure *C*. *deuterogattii* ST 173 at D7. The strain content changed again to *C*. *deuterogattii* ST 173 and *C*. *neoformans x C*. *deneoformans* hybrid content at D14 to return to a pure *C*. *deuterogattii* ST 173 infection at D28. Finally, the infection in patient 1 consisted of 3 different species in the same sample: *C*. *deuterogattii* ST 173, *C*. *neoformans* ST 5 and *C*. *neoformans x C*. *deneoformans* hybrid concurrently at W10. Interestingly, for this patient, *C*. *deuterogattii* ST 173 was persistent during the whole length of the follow-up. It is also worth noting that the *C*. *neoformans* ST 5 strain present early at D0, was not detected between D7 and D28 and was identified again at the W10 follow up point. Patients 3, 4 and 8 also showed a shift in content from D0 to D7. Patient 3’s infection shifted from *C*. *neoformans* ST 6 to *C*. *deuterogattii* ST 173. Patient 4’s infection strain composition went from full *C*. *neoformans x C*. *deneoformans* hybrid content at D0 to the occurrence of *C*. *neoformans* ST 93 strains alongside the hybrid at D7 and changed again to a pure *C*. *neoformans* ST 23 at D28. The infection of patient 8 went from *C*. *neoformans* ST 40 and ST 207 infection to a *C*. *neoformans* ST 40 and ST 555 infection (Fig 1).

### Antifungal susceptibility testing

Fig 1 and Table 2 summarize the *in vitro* susceptibility data for fluconazole and flucytosine obtained from the 252 clinical strains using the broth microdilution method, according to the CLSI M27-A3 protocol.

**Table 2:** Distribution of the 252 strains according to their MICs to Fluconazole, Flucytosine and Amphotericin B.

Antifungal Agent	Number of isolates with MIC (μg mL^-1^)													MIC50 (μg mL^-1^)	MIC90 (μg mL^-1^)	GM (μg mL^-1^)
	**0.09**	**0.06**	**0.125**	**0.25**	**0.5**	**1**	**2**	**4**	**8**	**16**	**32**	**64**	**NA**			
**Fluconazole**			31	29	48	41	34	28	9	16	15	1	1	[0,5–1]	[8–16]	1,14
**Flucytosine**			29	21	24	63	61	2	31	9	1	9	1	[0,5–1]	[3–4]	1,18
**Amphotericin B**	1		6	56	163	26								[0,25–0,5]	[0,5–1]	1

MIC to antifungals is indicated in μg mL^-1^

NA indicates strains for which MIC value was not available

MIC50 and MIC90.represent the concentration capable of inhibiting the growth of 50% and 90% of the isolates, respectively. GM represents the Geometric Mean

All isolates had an MIC ≤ 1 μg mL^-1^ to amphotericin B (Table 2 and S3 Table), with a geometric mean equal to 1 μg mL^-1^.

A total of 243/252 for flucytosine, (Fig 1 and Table 2). These strains (96.4%) had a wild-type phenotype. Nine (9/252; 3.6%) non-wild type strains with an MIC > 64 μg mL^-1^ were observed. Four out of nine isolates were hybrid *C*. *neoformans/C*. *deneoformans* types, 2/9 were *C*. *deuterogattii*, and 3/9 were *C*. *neoformans*. Six of the nine strains came from the same patient. The geometric mean for these clinical strains was equal to 1.18 μg mL^-1^ (Table 2).

Concerning fluconazole, 166/171 (97.1%) *C*. *neoformans* serotype A isolates had MICs ≤ 8 μg mL^-1^ and thus were wild type phenotype. Five isolates out of 171 (2.9%) had a MIC between 16 and 32 μg mL^-1^ so a none-wild type phenotype. They were all isolated from the same patient (Patient 11, W20). For the *C*. *deuterogatii* serotype B isolates, 41/42 (97.6%) had a wild-type phenotype with a MICs ≤ 32 μg mL^-1^ and 1/42 (2.4%) had a none-wild type phenotype with MIC > 64 μg mL^-1^(Fig 1). Finally, for the *C*. *neoformans x C*. *deneoformans* serotype AD hybrids, all proved to be of wild type phenotype with a MIC ≤ 16 μg mL^-1^.

Global geometric mean for fluconazole was equal to 1.14 μg mL^-1^ (Table 2).

## Discussion

Genetic diversity and analyses of susceptibility to antifungals of *Cryptococcus neoformans*/*Cryptococcus gattii* species from serial patient series are usually performed on a single isolate or well isolated single yeast colony by sample. The rationale behind this method is that serial isolates from the same patients belong mainly to the same genotype [46–48]. However, this approach harbours the risk of losing information and missing mixed infections. Moreover, *Cryptococcus* genetic diversity data are limited in sub-Saharan Africa, from which very few strains have been isolated and characterized.

In this study, the authors decided to apply a multiple isolate analysis (the whole initial culture grown from the CSF sample plus 5 randomly selected colonies) for each sample in order to obtain the largest overview of cryptococcal diversity in the cohort of Ivoirian patients.

First, the species/serotype distribution was analysed, and *C*. *neoformans* (serotype A) represented the majority of the isolated strains. This finding is in accordance with most epidemiological studies across the world. Indeed, *C*.*neoformans* serotype A forms the majority (72.5 to 96%) of the isolates identified whether in Africa [29, 49], China [50–52], Brazil [53] or Europe [17, 54–56]. A significant number of *C*. *deuterogattii* serotype B or *C*. *neoformans x C*. *deneoformans* hybrids serotype AD were also found in this study. In comparison, in most other studies, fewer AD hybrids and fewer serotype B were detected [17, 29, 49–52, 54–56]. These differences may be due to the originality of the sampling strategy in the present study. The serial sampling of patients combined with the analyses of multiple isolates per patient may have allowed us to highlight minor strains hidden in the background during mixed infections cases, which in turn may explain the higher proportion of *C*. *deuterogattii* serotype B and *C*. *neoformans x C*. *deneoformans* AD hybrids found here.

Comparison of this study population to the overall described STs showed two different situations for *C*. *neoformans* and *C*. *deuterogattii* species. In the *C*. *neoformans* group, two different genotypes, VNI with 5 different STs (ST 5, 6 23, 93 and 311) and VNII with 3 different STs (ST 40, 207 and 555), were grouped into two separate clonal complexes, one for each genotype. Thus, the *C*. *neoformans* population was clonal. Studies referencing the VNI genotype and related ST dispersal are abundant, with the STs found in this study showing a global dispersal. The ST 5 was the dominant ST found in this study. This ST, reported worldwide [19, 45, 51, 57–66], is frequently isolated from clinical samples but also from environmental and veterinary samples [58–60]. Its highest frequencies of detection occur in Asia. In Africa, ST 5 was isolated with variable rates depending on the study [19, 45]. Others STs such as ST 6 and ST 23 have been reported in America (North and South), Asia, Europe and Africa, [45, 57, 62, 64, 67]. ST 93 is frequently described in Asia and South America [48, 57, 62, 68] but seems uncommon in Africa [62]. To date, ST 311 has only been identified in Brazil [69] in both clinical and environmental samples. Finally, ST 23 was identified in South Africa [45] and Uganda [23] as well as in the present study.

The *C*. *deuterogattii* (VGII) population was more homogeneous than the *C*. *neoformans* population since all strains belonged to the same ST 173. On the contrary to *C*. *neoformans* VNI related ST, numerous *C*. *deuterogattii* STs seem to be linked specifically to a given geographical region [70–72]. Interestingly, ST 173 was first isolated in only one study from six patients, among whom five were immigrants from Africa to France and one was a resident of Senegal (http://mlst.mycologylab.org/Biolomics.aspx?Table=Sequence%20types%20C.%20gattii) [73]. Our results from Ivory Coast strengthen the hypothesis that ST 173 may be of African origin even if it could become more widely distributed in the future due to population movements. STs for *C*.*neoformans x C*. *deneoformans* hybrids were not determined because of unreadable profiles with the traditional MLST method. New methods to discriminate the hybrids as well as for identifying mixed infections in a single sample described by Chen *et al*. would be valuable in this situation [74,75].

Concerning the strain diversity in each patient, data in the literature vary based on the methods used. Desnos et al., 2010 [65], for example, recovered mixed cryptococcal infections in up to 18.4% of patients (9/49) by analysing from 4 to 33 single colonies/patients by serotyping and mating-type-specific locus PCR amplification. Previous studies with minisatellite and microsatellite amplifications showed higher levels of mixed infections ranging from 39% to 42% [30, 31] in series without follow-up when analysing 6 isolates per patient. Tomazin et al., 2018 also reported mixed infections within the same patient with serial series and sampling from 2 to 9 isolates per patient either at species, microsatellite genotype or AFLP fingerprinting levels [76]. In the present study, with serial sampling and analysis of 6 isolates, a majority of patients had constant strain content during their available follow-up period, while 4/13 patients were found with mixed species, mixed genotypes or mixed ST infections. Interestingly, two of the patients with constant strain content (patients 6 and 11) showed negative CSF during their follow-up before a relapse of CM with the same strains isolated before the negative culture. Whether the relapse was due to reactivation of the infection rendered dormant due to partially successful antifungal treatment [77] or a reinfection with strains more commonly present in the environment is, however, unclear.

Among the patients with mixed infections, patient 1 showed the highest number of different species by sample with up to 3 different species (*C*. *deuterogattii/C*. *neoformans/C*. *neoformans x C*. *deneoformans* hybrid). Such a number of species during mixed infection in the same patient and the same sample has not been described in the literature thus far. Patient 1 also experienced the most numerous switches in ST content. It is interesting to note that some strains present in the initial sampling (*C*. *neoformans* ST 5) were lost to detection, only to be found again at the last sampling time point, ten weeks later. This observation could represent a case of reinfection as the time delay of ten weeks between the loss and re-discovery of this ST is consistent with its definition in literature [49]. However, a relapse of infection caused by the same strain, hidden because of population shift during the course of treatment, cannot be excluded as it was shown that a same strain can persist into a patient for over 100 days [78].

Patient 3, in contrast, did not show a mixed infection in the same sample series but between series with a species content change (*C*. *neoformans* to *C*. *deuterogattii*) during the first week of follow-up. This short timeframe suggests that it is unlikely to be due to reinfection. In contrast, that patient may have been simultaneously infected by both strains with *C*. *deuterogattii* later overwhelming *C*. *neoformans*. Indeed, the high dose of FCZ treatment started on inclusion may have allowed the emergence of *C*. *deuterogattii* yeast due to the lower susceptibility of this species to the antifungal [79,80].

Patient 4 experienced a progressive change in strain content with a full *C*. *neoformans x deneoformans* hybrid population on inclusion, turning into a mixed hybrid and *C*. *neoformans* ST 93 after one week and ending into 100% *C*. *neoformans* ST 23, 3 weeks later. This strain change could be explained by two hypotheses: either the *C*. *neoformans x deneoformans* hybrid and ST 93 isolate became significantly reduced in quantity over time in regard to the emerging ST 23 isolates and were missed during cloning at W10, or the initial high dose of fluconazole (1600 mg/day) in the first week of the protocol helped to eliminate the early strains, leaving an available ecological niche for the emergence of the ST23 on late stages.

Finally, patient 8 did not show any mixed infection at the species level. However, he presented a change in STs during his first week of follow-up with VNI ST40 and VNI ST 207 turning into VNI ST40 and VNI ST 555. ST 207 and ST 555 are included in the same clonal complex but differ by only a single nucleotide in the GPD1 sequence. All these results confirm that the analysis of several isolates for each patient sample allows to report a diversity possibly masked by an isolate having a better fitness or over-represented in the initial sample. This sampling approach limits the loss of information regarding minor strains. The largest assessment of cryptococcal genetic diversity is important because it was shown for other yeast, *i*.*e*., *Candida sp*., that mixed infections could lead to treatment complications or failures [81–83] and to the emergence of species or strains resistant to antifungal drugs [84,85].

Finally, susceptibility to fluconazole, flucytosine and amphotericin B was analysed for the 252 isolates. All strains proved to be susceptible to amphotericin B. Very few none-wild type phenotypes to flucytosine were found and numbers were in accordance with previous studies in in Africa [86] and Asia [87]. Concerning fluconazole, very few none-wild type strains were detected either. The overall resistance level to fluconazole is thus lower than what can be found in previous studies in Cameroon [33], Kenya [88], Uganda [89] and South Africa [90]. It is also 3 time lower than the mean resistance rate of 12.4% assessed for the whole African region [91,92] It was proposed that this high resistance rate in Africa may have been due to limited access to amphotericin B, flucytosine or ARTs and to the use of fluconazole in low-dose monotherapy as first-line therapy [93]. Thus, the low resistance level found in the present study could be explained by: i) the high dose fluconazole protocol based on previous empirical trials [94–96]. ii) the fluconazole-flucytosine combination, shown to limit the amplification of resistance [97] iii) the monitoring of the patients to ensure they took their ARV and antifungal treatments correctly. iv) the variation in ECVs between ancient studies [88–90] and more recent recommendations, especially for *C*. *gattii* [44].

Because of the high dose of FCZ in the initial treatment followed by lower doses over 20 weeks, MIC increases between the initial and follow-up samples in the same patient were expected [98–100]. It is known that *in vitro* growth of *C*. *neoformans* in the presence of sub lethal concentrations of FCZ induces the selection of resistant colonies with elevated MICs to FCZ [101,102], and a similar increase was shown to occur in infected mice that were treated with FCZ [103]. In this study, no significant increase in the MIC to FCZ was observed over time possibly thanks to the fluconazole-flucytosine combination protocol [97]. However, the sampling strategy allowed us to show that the coexistence of a mix of wild-type and none-wild type strains was possible in the same patient (as described elsewhere [33]) and same sample. In such cases, the MIC ranges showed variations up to 5 dilutions. These variations were observed for isolates including those showing the same sequence type. No correlation between *Cryptococcus neoformans* VNI and VNII STs, or *Cryptococcus deuterogattii* VGII ST and an elevated MIC to FCZ was found.

In conclusion, this study showed that mixed infections could be identified at the species level down to the sequence type level, as well as at the susceptibility to antifungal level in the same patients over time. Up to 3 different species were found alongside up to 4 different STs in the same patient. This diversity could be due to reinfections from nearby environmental strains during follow-up, the emergence of minor populations due to antifungal pressure, degradation of patient health or genetic microevolution of the strains. This study provides new data on the *Cryptococcus* epidemiology in West Africa and Ivory Coast and shows the complexity of the evolution of a cryptococcal population in a pool of patients as well as the various mechanisms leading to this evolution.

## Supporting information

S1 Table Demographic characteristics of the patients and outcome of infection. (DOCX)

S2 Table Distribution of the Cryptococcus serotypes, genotypes, STs and MICs to antifungal ranges by patients throughout the whole follow up. MICs ranges to antifungals are expressed in μg mL^-1^.
(DOCX)

S3 Table Demographic characteristics of the patients, sero-genotyping, ST characterization and MIC (μg mL-1) to amphotericin B for the totality of the strains isolated during the follow up. (a) Indicate a depletive spinal tap; (b) indicate patient sampled at W9, dead at W10; (c) indicate spinal fluid positive, culture negative in Ivory Coast, positive in Montpellier, (d) indicate a patient sampled outside ANRS protocol because of relapse at W24, (e) indicate a patient sampled outside ANRS protocol with a discharge spinal tap at W26, (f) indicate a patient sampled at D10, death at D14, (g) and light grey indicate negative spinal fluid and cultures, (h) indicate positive spinal fluid, cultures positives in Ivory Coast negative in Montpellier, (i) and middle grey indicate a patient lost to follow up, (j) and strong grey indicate a deceased patient. ND shows undetermined ST. The red bar shows the ANRS 12257 Study Endpoint.
(TIF)

S1 Fig Minimum Spanning Tree showing distribution of the isolates found in Ivorian patients from this study and compared to the global diversity described for C. neoformans in literature. The figure shows the distribution of the isolates found in the present study when compared to the global diversity of 488 ST forming 38 CC and 182 singletons described for *C*. *neoformans*. ST forming CC including all the VN I isolates or including all the VN II isolates found in the present study are surrounded in red and green respectively. The ST in grey are shown the ST described in literature but not found in this study.
(PDF)
