# Supplementary material for: Cryptococcus genetic diversity and mixed infections in Ivorian HIV patients: A follow up study
Source: PLoS Negl Trop Dis. 2019 Nov 18;13(11):e0007812. doi: 10.1371/journal.pntd.0007812 (PMC6886875; doi:10.1371/journal.pntd.0007812)
Supplement: S1 Table — (DOCX) [file pntd.0007812.s001.docx]

|  | Demographic characteristics of the patients and outcome | | | | | | | | | | | | |
| --- | --- | --- | --- | --- | --- | --- | --- | --- | --- | --- | --- | --- | --- |
| Patient number | 1 | 2 | 3 | 4 | 5 | 6 | 7 | 8 | 9 | 10 | 11 | 12 | 13 |
| Age | 40 | 37 | 39 | 39 | 41 | 60 | 39 | 46 | 35 | 43 | 42 | 43 | 56 |
| Sexe | F | F | F | M | M | F | F | M | F | M | F | M | M |
| HIV Type | 1 | 1 | 1 | 1 | 1 | 1 and 2 | 1 | 1 | 1 | 1 | 1 | 1 | 1 |
| Outcome | Death (W10) | Death (between W10 and W24) | Alive | Alive | Alive | Alive | Death (before D20) | Alive | (Death (after W10) | Death (after W10) | Alive | Death (before D14) | Death (before D14) |
