# Supplementary figures and images for: Cryptococcus genetic diversity and mixed infections in Ivorian HIV patients: A follow up study
Source: PLoS Negl Trop Dis. 2019 Nov 18;13(11):e0007812. doi: 10.1371/journal.pntd.0007812 (PMC6886875; doi:10.1371/journal.pntd.0007812)

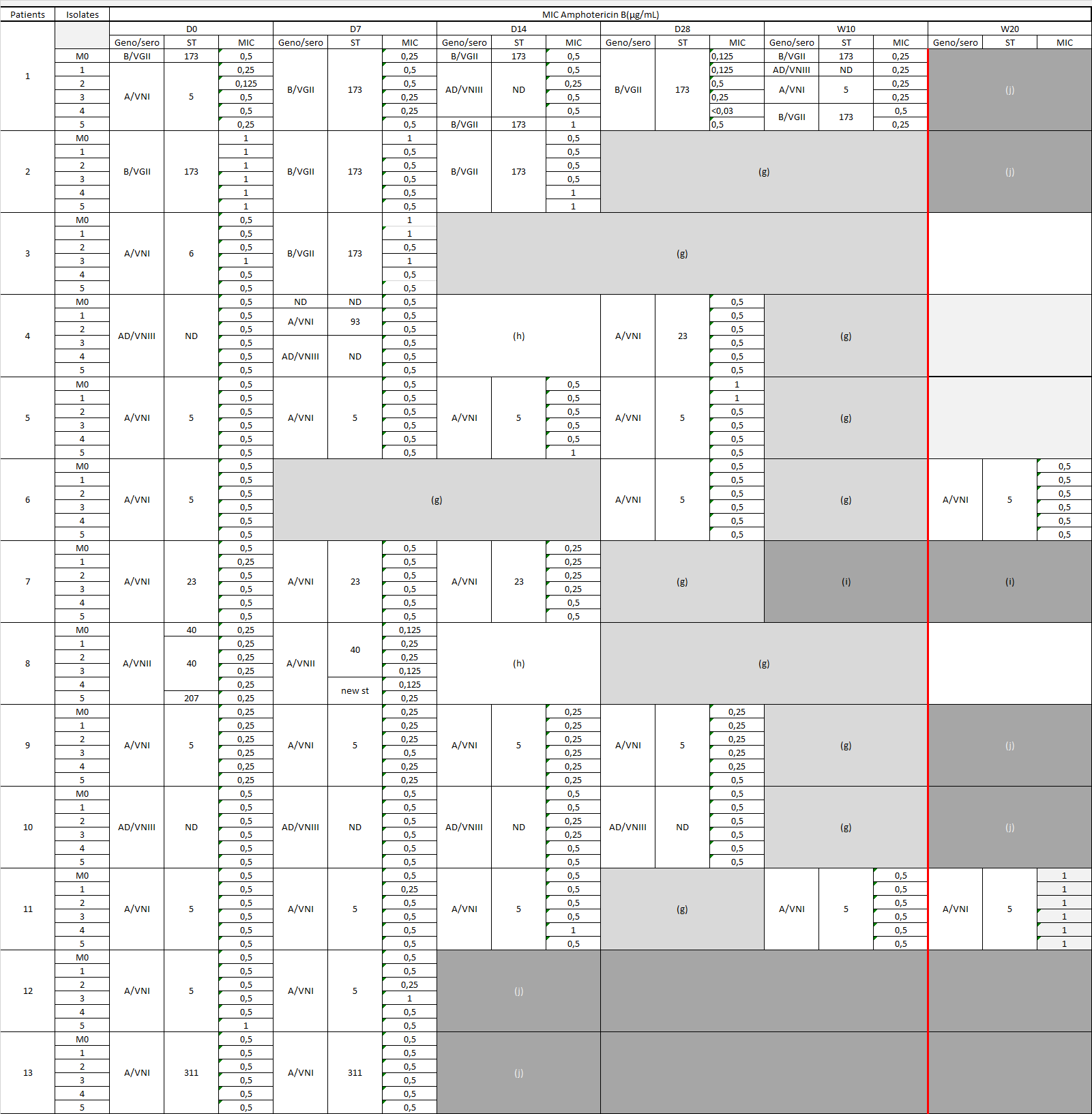

Supplement: S3 Table — (a) Indicate a depletive spinal tap; (b) indicate patient sampled at W9, dead at W10; (c) indicate spinal fluid positive, culture negative in Ivory Coast, positive in Montpellier, (d) indicate a patient sampled outside ANRS protocol because of relapse at W24, (e) indicate a patient sampled outside ANRS protocol with a discharge spinal tap at W26, (f) indicate a patient sampled at D10, death at D14, (g) and light grey indicate negative spinal fluid and cultures, (h) indicate positive spinal fluid, cultures positives in Ivory Coast negative in Montpellier, (i) and middle grey indicate a patient lost to follow up, (j) and strong grey indicate a deceased patient. ND shows undetermined ST. The red bar shows the ANRS 12257 Study Endpoint. (TIF) [file pntd.0007812.s003.tif]

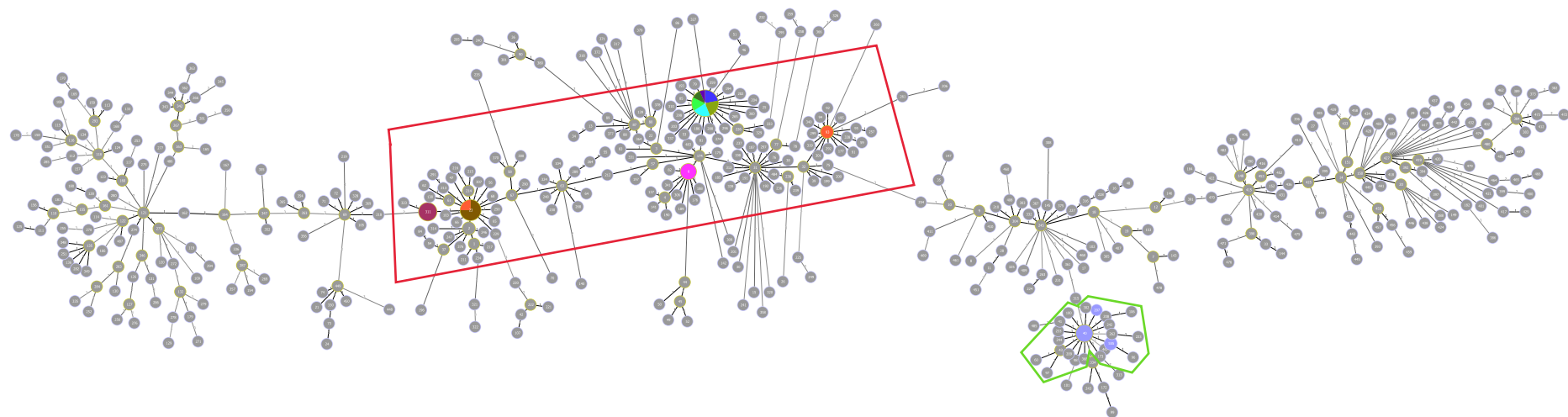

Supplement: S1 Fig — The figure shows the distribution of the isolates found in the present study when compared to the global diversity of 488 ST forming 38 CC and 182 singletons described for C. neoformans. ST forming CC including all the VN I isolates or including all the VN II isolates found in the present study are surrounded in red and green respectively. The ST in grey are shown the ST described in literature but not found in this study. (PDF) [file pntd.0007812.s004.pdf]
