# Supplementary material for: Cryptococcus genetic diversity and mixed infections in Ivorian HIV patients: A follow up study
Source: PLoS Negl Trop Dis. 2019 Nov 18;13(11):e0007812. doi: 10.1371/journal.pntd.0007812 (PMC6886875; doi:10.1371/journal.pntd.0007812)
Supplement: Supplementary file 3 [file pntd.0007812.s002.docx]

				Range (µg mL^{-1})		
**Patients**	**Serotype**	**Genotype**	**ST**	**FCZ**	**5FC**	**AMB**
1	A, B, AD	VNI, VGII, VNIII	5, 173, ND	[0,5-8]	[0,5-64]	[0,125-0,5]
2	B	VGII	173	[8-64]	[0,25-8]	[0,5-1]
3	A, B	VNI, VGII	6, 173	[0,125-32]	[0,25-8]	[0,5-1]
4	A, AD	VNI, VNIII	93, ND, 23	[0,125-4]	[0,125-8]	0,5
5	A	VNI	5	[0,5-2]	[0,25-4]	[0,5-1]
6	A	VNI	5	[0,125-4]	[0,25-4]	0,5
7	A	VNI	23	[0,125-1]	[0,125-2]	[0,25-0,5]
8	A	VNII	40, 207, 555	[0,25-0,5]	[0,25-1]	[0,125-0,25]
9	A	VNI	5	[0,25-0,5]	[0,5-4]	[0,25-0,5]
10	AD	VNIII	ND	[0,125-4]	[0,125-8]	[0,25-0,5]
11	A	VNI	5	[0,125-32]	[0,125-64]	[0,25-1]
12	A	VNI	5	[0,125-2]	[0,125-16]	[0,25-1]
13	A	VNI	311	[0,125-1]	[0,25-2]	0,5
